# CzcR Is Essential for Swimming Motility in Pseudomonas aeruginosa during Zinc Stress

**DOI:** 10.1128/spectrum.02846-22

**Published:** 2022-11-23

**Authors:** Zhiqing Liu, Zirui Xu, Shuzhen Chen, Jiahui Huang, Ting Li, Cheng Duan, Lian-Hui Zhang, Zeling Xu

**Affiliations:** a Guangdong Province Key Laboratory of Microbial Signals and Disease Control, Integrative Microbiology Research Centre, South China Agricultural University, Guangzhou, People’s Republic of China; b Guangdong Laboratory for Lingnan Modern Agriculture, South China Agricultural University, Guangzhou, People’s Republic of China; South China Sea Institute of Oceanology

**Keywords:** *Pseudomonas aeruginosa*, TCS, CzcR, swimming, zinc stress

## Abstract

Two-component system (TCS) plays a vital role in modulating target gene expression in response to the changing environments. Pseudomonas aeruginosa is a ubiquitous opportunistic pathogen that can survive under diverse stress conditions. The great adaptability of P. aeruginosa relies heavily on the abundant TCSs encoded by its genome. However, most TCSs in P. aeruginosa have not been well-characterized. CzcS/CzcR is a metal responsive TCS which displays multiple regulatory functions associated with metal hemostasis, quorum sensing activity and antibiotic resistance. In this study, we found that swimming motility of P. aeruginosa was completely abolished during zinc (Zn^2+^) stress when the *czcR* gene from the TCS CzcS/CzcR was deleted. Noticeably, CzcR was dispensable for swimming without the stress of Zn^2+^ excess. CzcR was shown to be activated by Zn^2+^ stress possibly through inducing its expression level and triggering its phosphorylation to positively regulate swimming which was abolished by Zn^2+^ stress in a CzcR-independent manner. Further TEM analyses and promoter activity examinations revealed that CzcR was required for the expression of genes involved in flagellar biosynthesis during Zn^2+^ stress. In vitro protein-DNA interaction assay showed that CzcR was capable of specifically recognizing and binding to the promoters of operons *flgBCDE*, *flgFGHIJK,* and *PA1442/FliMNOPQR/flhB*. Together, this study demonstrated a novel function of CzcR in regulating flagellar gene expression and motility in P. aeruginosa when the pathogen encounters Zn^2+^ stress conditions.

**IMPORTANCE** The fitness of bacterial cells depends largely on their ability to sense and respond quickly to the changing environments. P. aeruginosa expresses a great number of signal sensing and transduction systems that enable the pathogen to grow and survive under diverse stress conditions and cause serious infections at different sites in many hosts. In addition to the previously characterized functions to regulate metal homeostasis, quorum sensing activity, and antibiotic resistance, here we report that CzcR is a novel regulator essential for flagellar gene expression and swimming motility in P. aeruginosa during Zn^2+^ stress. Since swimming motility is important for the virulence of P. aeruginosa, findings in this study might provide a new target for the treatment of P. aeruginosa infections with Zn^2+^-based antimicrobial agents in the future.

## INTRODUCTION

Bacteria frequently encounter a wide variety of challenges from ever-changing environmental stresses such as the shift of temperature, pH, metal concentration, nutrient availability, and antimicrobial threat. Rapid reprograming of gene expression profiles and adapting to various environmental stresses are crucial for bacteria to survive, which relies on the cooperation of abundant regulatory systems and complicated signal transduction networks in the cell (1). Among them, two-component system (TCS) represents one of the most ubiquitous mechanisms employed by bacteria to sense environmental stresses and control physiological behaviors accordingly (2). An increasing number of studies have reported that many TCSs are indispensable for the expression of genes associated with virulence and antibiotic resistance (3, 4). Thus, TCSs have been recognized as a group of important targets for antimicrobial therapies (5).

A typical TCS is composed of a membrane-bound sensor histidine kinase (HK) that detects specific signals and a cognate response regulator (RR) which regulates downstream target gene expression (6). A typical HK is a homodimeric integral membrane protein that contains two transmembrane (TM) helices flanking a sensor domain in the periplasmic space and one of the TM helices connected a HAMP (commonly found in Histidine kinases, Adenylyl cyclases, Methyl-accepting chemotaxis proteins, and Phosphatases) domain, a DHp (dimerization and histidine phosphorylation) domain, and a C-terminal CA (catalytic and ATP-binding) domain in the cytosol (7). RR generally contains a REC receiver domain and a linked effector domain (8). When the HK detects a specific environmental stimulus, signal transduction occurs through the ATP-dependent phosphorylation of a histidine (H) residue in the DHp domain by the CA domain. Subsequently, the REC domain in RR catalyzes the transfer of the phosphoryl group from the HK to its aspartate (D) residues, triggering the conformational changes of RR, and leading to activation of the linked effector domain to modulate downstream gene expression (6).

Pseudomonas aeruginosa is a ubiquitous environmental bacterium commonly found in soil and water (9). It is also an opportunistic human pathogen that can produce numerous virulence factors to cause nosocomial acute and chronic infections (10). Genome analysis indicated that P. aeruginosa encodes abundant TCSs. There are at least 64 HKs and 72 RRs expressed by the PAO1 genome (11, 12). Although a lot of them remain uncharacterized, studies have shown the importance of TCSs in regulating many cellular behaviors related to its pathogenicity and antibiotic resistance in P. aeruginosa in response to diverse environmental cues (4, 13). For instance, PhoR/PhoB senses inorganic phosphate to regulate the production of quorum-sensing-controlled virulence factors such as pyocyanin (PYO) (14). CbrA/CbrB senses various carbon sources and modulates motility, biofilm formation, and antibiotic resistance (15).

Zinc (Zn^2+^) is the second most abundant transition metal ion in bacteria and serves as an important cofactor in many enzymatic reactions while excessive Zn^2+^ is cytotoxic. P. aeruginosa has developed a delicate Zn^2+^ homeostatic network to support its growth under Zn^2+^-depleted and high Zn^2+^ conditions by coordinating the processes of Zn^2+^ uptake, storage, and extrusion (16). CzcS/CzcR was previously identified as a Zn^2+^-responsive TCS, which plays an essential role in maintaining homeostasis and elevating tolerance of Zn^2+^ and other metal ions such as cadmium and cobalt by upregulating the expression of the efflux system CzcCBA (17–19). The response regulator CzcR in P. aeruginosa was also found to repress the expression of OprD, which is a porin for the entry of carbapenem antibiotics and therefore induces the recalcitrance of P. aeruginosa to these antibiotics (17, 20). Moreover, CzcR was reported to mediate the repression of PYO production and biofilm formation in the presence of excessive Zn^2+^ or ZnO nanoparticles (21). Further experiments demonstrated that CzcR modulates quorum sensing and antibiotic resistance by directly binding with the promoters of genes such as *lasI*, *phzA1,* and *oprD* (20). These findings exhibited the versatility of CzcS/CzcR to control both virulence and antibiotic resistance in P. aeruginosa. However, it remains unexplored whether CzcS/CzcR plays additional regulatory roles and participates in other physiological processes.

Flagellum-dependent swimming motility is known as an important virulence factor in P. aeruginosa for the pathogen to rapidly disseminate from initial infection sites to different tissues and establish biofilms to cause life-threatening infections (22–26). Studies have revealed that P. aeruginosa has complicated regulatory systems, including several TCSs such as PilS/PilR and FleS/FleR, to modulate flagellar biosynthesis and swimming motility (27, 28). In the present study, we showed that CzcS/CzcR is essential for flagellar gene expression and swimming motility in P. aeruginosa during Zn^2+^ stress. Deletion of *czcR* in P. aeruginosa completely inactivates its swimming motility during Zn^2+^ stress. Findings in this study not only expanded our understanding in the regulon of CzcS/CzcR in P. aeruginosa but also provided implications for antipseudomonal therapies by targeting this TCS.

## RESULTS

### CzcR regulated PYO production and swimming motility in the presence of Zn^2+^ stress.

**FIG 1:**
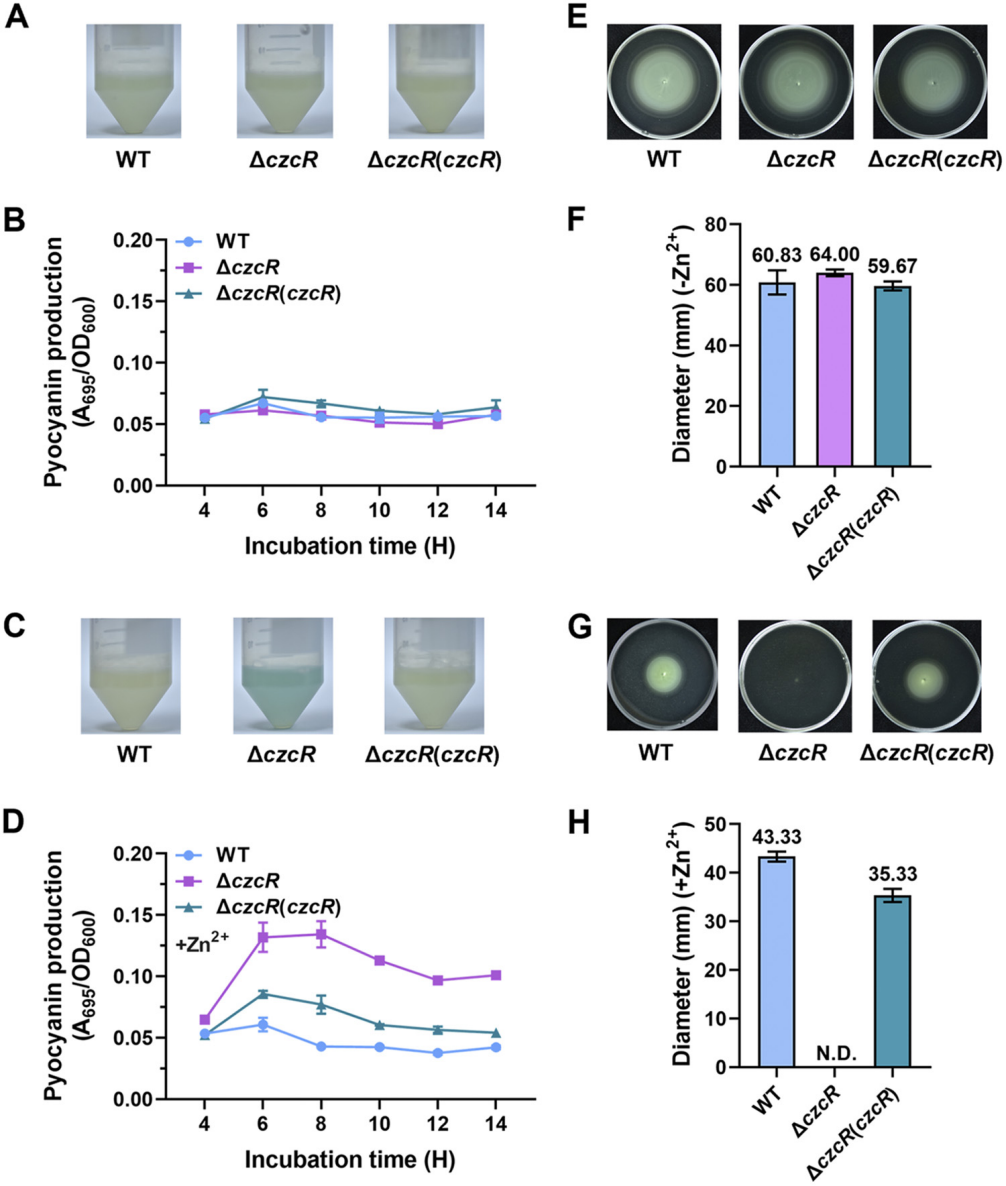
CzcR negatively regulates PYO biosynthesis and positively regulates swimming motility in the presence of Zn^2+^ stress. (A) Liquid culture of the strains of PAO1 WT, Δ*czcR,* and Δ*czcR* with complemented expression of *czcR* (Δ*czcR[czcR]*) when they were grown without the supplementation of Zn^2+^. (B) Quantification of PYO production at different time points during growth without the supplementation of Zn^2+^ in PAO1 WT, Δ*czcR,* and Δ*czcR*(*czcR*) strains. (C) Liquid culture of the PAO1 WT, Δ*czcR,* and Δ*czcR*(*czcR*) strains grown with the supplementation of 0.5 mM Zn^2+^. (D) Quantification of PYO production at different time points during growth with the supplementation of 0.5 mM Zn^2+^ in PAO1 WT, Δ*czcR,* and Δ*czcR*(*czcR*) strains. (E) Examination of swimming motility of the PAO1 WT, Δ*czcR,* and Δ*czcR*(*czcR*) strains on the plates without the supplementation of Zn^2+^. (F) Diameters of the swimming zone of the PAO1 WT, Δ*czcR*, and Δ*czcR*(*czcR*) strains grown without the supplementation of Zn^2+^. (G) Examination of swimming motility of the PAO1 WT, Δ*czcR*, and Δ*czcR*(*czcR*) strains on the plates with the supplementation of 0.5 mM Zn^2+^. (H) Diameters of the swimming zone of the PAO1 WT, Δ*czcR*, and Δ*czcR*(*czcR*) strains grown with the supplementation of 0.5 mM Zn^2+^. N.D., not detected.

To explore physiological functions of the TCS CzcS/CzcR, we deleted the *czcR* gene and attempted to detect the phenotypical differences between the Δ*czcR* mutant and the PAO1 wild-type (WT) strain. Because PYO is a kind of blue/green pigment, its production level can be easily evaluated with the color of the liquid culture. However, in contrast to the previous report that deletion of the *czcR* gene in PAO1 led to a substantial accumulation of PYO (20), we did not observe any difference between the WT and Δ*czcR* strains when they were cultured in the liquid LB medium (Fig. 1A). PYO quantifications for the WT strain, Δ*czcR* mutant and the Δ*czcR* mutant complemented with *czcR* at different time points during growth in the liquid medium did not show any difference in PYO production as well (Fig. 1B). The discrepant results led us to speculate that CzcR in the PAO1 WT strain was inactive because these two strains were cultured in normal LB medium without supplementing any inducible elements for this TCS system. We then treated these strains with a stressed concentration (0.5 mM) of ZnSO_4_ (Zn^2+^). A slight decrease of PYO production was observed in the PAO1 WT strain when it was grown in the presence of Zn^2+^ stress compared to the absence of Zn^2+^ stress (Fig. 1A and C), which was in accordance with the previous study showing the inhibition of PYO production by ZnO nanoparticles (21). Interestingly, in the presence of Zn^2+^ stress, we observed a substantial accumulation of the blue/green-colored PYO in the medium of the Δ*czcR* strain, which was greatly higher than that in the WT strain and could be reduced nearly back to the WT level when the mutant was complemented with *czcR* (Fig. 1C). PYO accumulation in the Δ*czcR* strain was not observed by supplementing Na_2_SO_4_ instead of ZnSO_4_ (Fig. S1A), confirming that it was Zn^2+^ that induced the production of PYO in CzcR-independent pathways while concurrently Zn^2+^ activated CzcR to antagonize the induction of PYO biosynthesis. Further quantifications of PYO at different time points during cell growth showed that deletion of *czcR* resulted in a substantially increased PYO production after 6-h incubation compared to the WT strain (Fig. 1D).

In addition to the liquid culture, we also noticed an obvious difference in the colony morphology between PAO1 WT and Δ*czcR* strains when they were streaked on the solid agar plates. As with the PYO production, no difference of the colony phenotypes was observed between the WT and Δ*czcR* strains without supplementation of Zn^2+^ in the agar plates, while, interestingly, most colonies of the Δ*czcR* strain became smoother than the WT strain when 0.5 mM Zn^2+^ was supplemented in the agar plates (Fig. S2A). This was more evident when the colony edge was enlarged under a stereo microscope (Fig. S2B). It was reported that bacterial motility is different between smooth and rough colonies in other species (29). Thus, we suspected that CzcR might play a role in regulating motility besides its already known functions. To test this speculation, we examined the swimming motility of the PAO1 WT, Δ*czcR*, and Δ*czcR*(*czcR*) strains. As shown in Fig. 1E, still no difference was observed between these strains without the presence of Zn^2+^ stress with regard to their swimming motility. Although Zn^2+^ stress reduced swimming motility of the PAO1 WT strain by decreasing its swimming zone from 60.83 mm to 43.33 mm, swimming motility was completely abolished when *czcR* was deleted during Zn^2+^ stress and complementation of *czcR* restored the swimming motility of Δ*czcR* (Fig. 1E to H). As a control, Na_2_SO_4_ did not cause any difference in the swimming motility between PAO1 WT and Δ*czcR* strains (Fig. S1B). These results indicated that CzcR was essential for P. aeruginosa swimming during Zn^2+^ stress. As with the regulatory pattern of PYO production, it seemed that the swimming motility was inhibited by Zn^2+^ stress through CzcR-independent pathways, but CzcR was activated in the presence of Zn^2+^ stress to concurrently rescue the inhibited swimming motility. We further monitored the survival of the PAO1 WT and Δ*czcR* strains in the absence and presence of Zn^2+^ stress, respectively, to ensure that loss of CzcR did not influence cell viability. The result showed that cell growth on the agar plates containing Zn^2+^ was not influenced by the deletion of *czcR* (Fig. S3), excluding the possibility that swimming motility of the Δ*czcR* strain was abolished owing to its defective growth during Zn^2+^ stress. Since CzcR inhibits PYO production by repressing the quorum sensing system and the PYO biosynthetic gene cluster *phz* as previously reported (20), we next continued to focus on exploring how swimming motility was controlled by CzcR during Zn^2+^ stress.

### D51 was a potential residue in CzcR for phosphorylation to regulate swimming motility.

**FIG 2:**
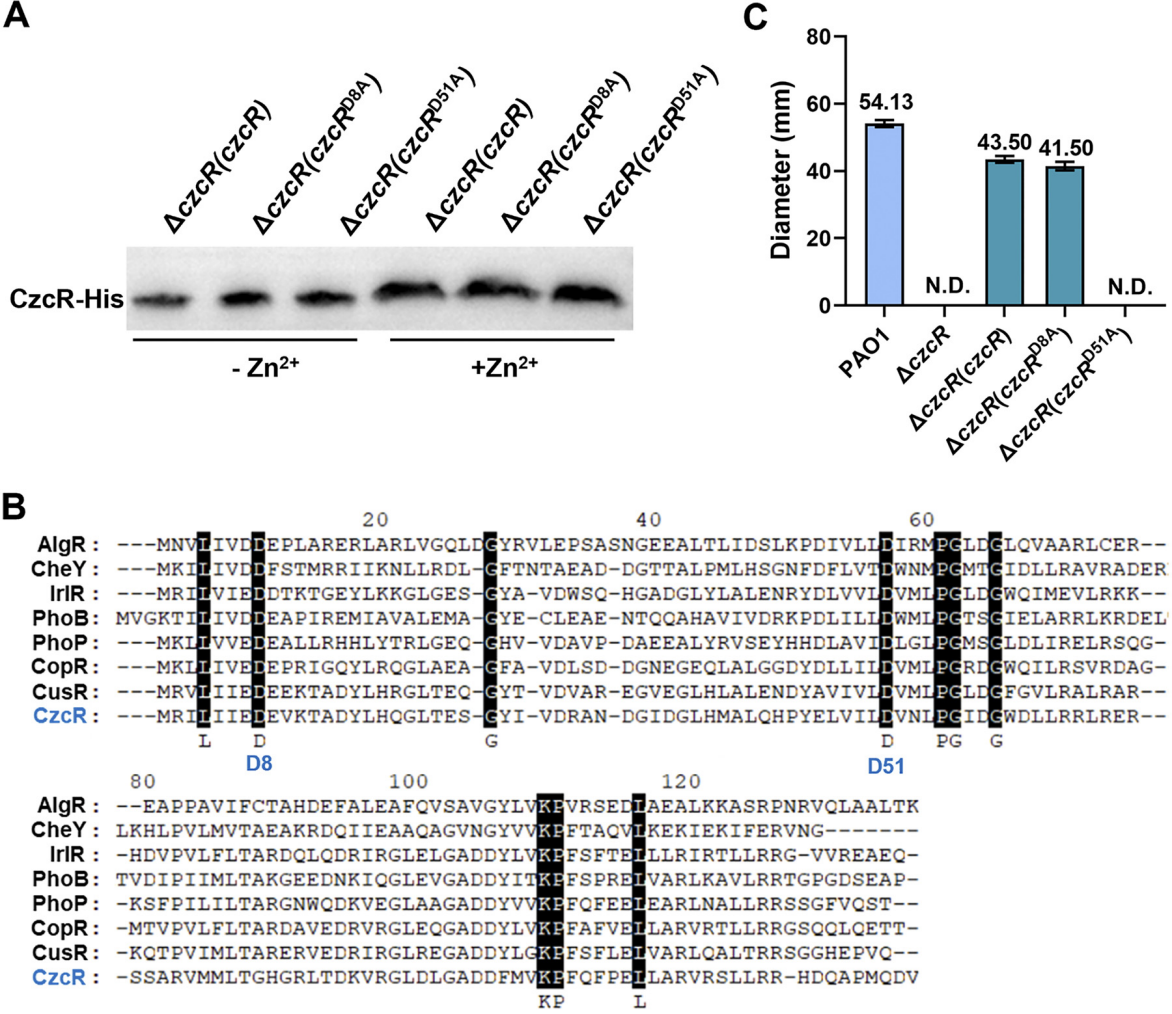
Zn^2+^ induces the expression of CzcR and D51 in CzcR is a key residue to regulate swimming motility. (A) Translational levels of the complemented CzcR or its variants with D81A and D51A point mutations in the Δ*czcR* mutant grown in the absence or presence of Zn^2+^. (B) Sequence alignment of the receiver domain of CzcR with other TCS response regulators. Conserved amino acid residues were highlighted with black background and two conserved aspartate residues D8 and D51 were indicated. (C) Examination of swimming motility of the PAO1 WT strain, Δ*czcR* mutant, and the Δ*czcR* mutant complemented with *czcR* or its D8A and D51A variants grown on the plates with the supplementation of Zn^2+^. N.D., not detected.

Because CzcR did not exhibit any regulatory function on PYO production and swimming motility in the absence of the inducible signal Zn^2+^, we speculated that CzcR requires Zn^2+^ to either promote its expression or induce its activity. Upregulated transcription of *czcR* by Zn^2+^ was previously reported (17). Here, we further demonstrated that presence of Zn^2+^ resulted in increased expression of CzcR at protein level by performing the Western blot assay (Fig. 2A). Given the signal transduction mode between the TCS pairs, we then attempted to investigate whether the regulatory function of the overexpressed CzcR to promote swimming motility was also associated with its phosphorylation status. Sequence alignment based on a set of well-characterized RRs using MEGA (version 11.0) led to the identification of two highly conserved aspartate residues, i.e., D8 and D51 (Fig. 2B) (30), which were potentially responsible for signal transduction by receiving the phosphoryl groups in CzcR. We therefore replaced these residues at D8 and D51 in the complemented CzcR with alanine (A) and tested the swimming motility of the Δ*czcR* mutant with these CzcR variants. It showed that replacement at the 8th position (D8A) did not affect the function of CzcR to promote swimming while replacement at the 51st position (D51A) completely prevented its activity to promote swimming without influencing the protein translation and stabilization (Fig. 2A and C). Consistent with this, D51 was the only aspartate residue detected in the conserved acidic pocket, which is the active site of RR containing the phosphorylatable aspartate residue using the ScanProsite tool (31). These results together suggested that the activity of CzcR was upregulated during Zn^2+^ stress and D51 was a key aspartate residue for CzcR to regulate swimming motility possibly serving as the residue for phosphorylation.

### CzcR activated the expression of genes involved in flagellar biosynthesis during Zn^2+^ stress.

**FIG 3:**
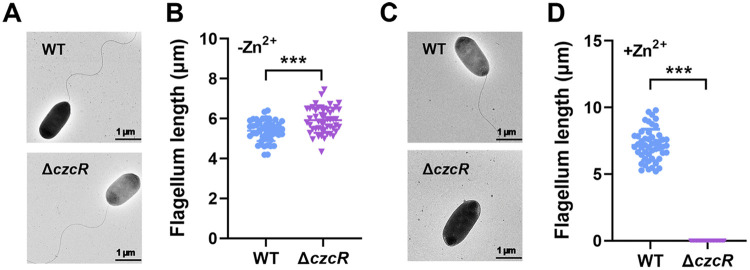
CzcR is required for flagellar biosynthesis during Zn^2+^ stress. (A) Observation of flagella in the PAO1 WT and Δ*czcR* strains grown in the absence of Zn^2+^ stress by TEM. Representative images for each sample were shown. (B) Flagellar length measured for the PAO1 WT and Δ*czcR* strains grown in the absence of Zn^2+^ stress. Fifty cells from each strain were selected for the measurement. (C) Observation of flagella in the PAO1 WT and Δ*czcR* strains grown in the presence of Zn^2+^ stress by TEM. (D) Flagellar length measured for the PAO1 WT and Δ*czcR* strains grown in the presence of Zn^2+^ stress. ***, *P < *0.001 compared to WT based on Student's *t* test.

Given that swimming motility is a type of unicellular movement behavior in liquid or low-viscosity conditions that requires a functional flagellum (32), we first investigated whether deletion of *czcR* affected flagellar biosynthesis during Zn^2+^ stress. A transmission electron microscope (TEM) was applied to observe the flagellar morphology of the PAO1 WT and Δ*czcR* strains after they were cultured in the presence and absence of Zn^2+^ stress. TEM micrographs showed that the flagellar morphology of PAO1 WT and Δ*czcR* cells were similar but with a slight increase of the average flagellar length in the Δ*czcR* cells when they were grown in the absence of Zn^2+^ stress (Fig. 3A and B). However, in the presence of Zn^2+^ stress, flagella were not observed in the Δ*czcR* cells, whereas they were still intact in the PAO1 WT strain as shown in Fig. 3C and D. Consistent with the swimming pattern, this result demonstrated that flagellar biosynthesis was inhibited by Zn^2+^ stress in a CzcR-independent manner, and CzcR was induced and activated to stimulate flagellar biosynthesis.

**FIG 4:**
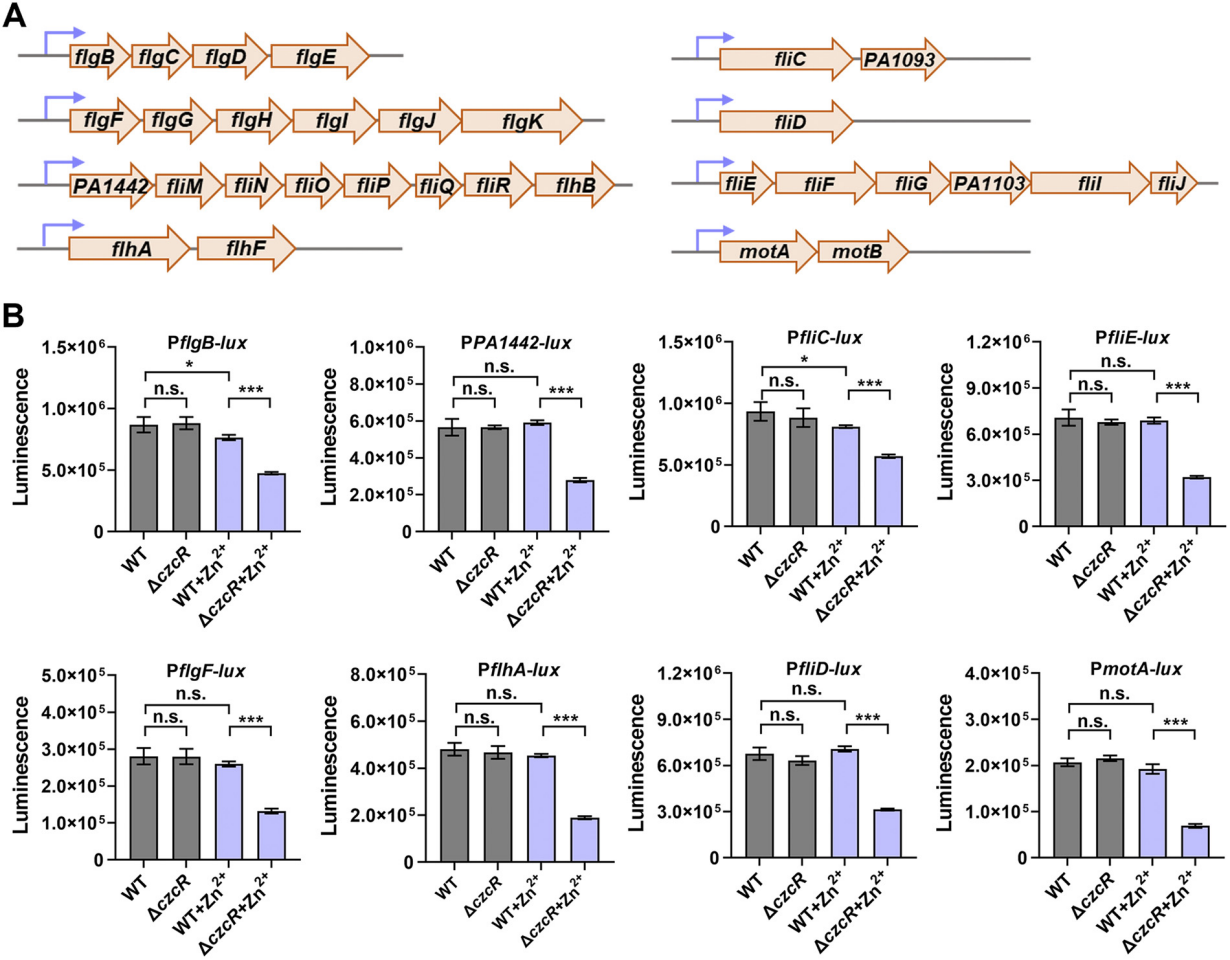
CzcR regulates the expression of genes involved in flagellar biosynthesis during Zn^2+^ stress. (A) Major genes or operons involved in flagellar biosynthesis in P. aeruginosa. (B) Promotor activities of genes or operons involved in flagellar biosynthesis in PAO1 WT and Δ*czcR* strains grown in the absence or presence of Zn^2+^ stress. n.s., not significant, *, *P < *0.05, ***, *P < *0.001 based on Student's *t* test.

P. aeruginosa flagellum is composed of three elements, including a membrane complex, a hook, and a flagellin filament (33). These compositions are mainly transcribed from the operons or genes of *flgBCDE*, *flgFGHIJKL*, *PA1442/FliMNOPQR/flhB*, *flhAF*, *fliC/PA1093*, *fliD*, *fliEFG/PA1103/fliIJ,* and *motAB* based on the annotations in the Pseudomonas Genome Database (https://www.pseudomonas.com/) (Fig. 4A) (34). To measure the expression of these operons or genes, promoter-*lux* transcriptional fusions were constructed for all these promoters and luminescence reporter assay was performed to evaluate their activity. As shown in Fig. 4B, all the promoters, i.e., P*_flgBCDE_* (P*flgB-lux*), P*_flgFGHIJKL_* (P*flgF-lux*), P*_PA1442/FliMNOPQR/flhB_* (P*PA1442-lux*), P*_flhAF_* (P*flhA-lux*), P*_fliC/PA1093_* (P*fliC-lux*), P*_fliD_* (P*fliD-lux*), P*_fliEFG/PA1103/fliIJ_* (P*fliE-lux*), and P*_motAB_* (P*motA-lux*), were significantly repressed with the deletion of *czcR* during Zn^2+^ stress, suggesting that the abolished swimming motility in Δ*czcR* during Zn^2+^ stress was associated with the repression of genes involved in flagellar biosynthesis. Consistent with the swimming pattern (Fig. 1E), deletion of *czcR* did not influence the transcriptional activity of these promoters in the absence of Zn^2+^ stress (Fig. 4B). These results implied that Zn^2+^ stress repressed the expression of genes involved in flagellar biosynthesis probably in a CzcR-independent manner, while CzcR was activated concurrently to rescue the expression of these genes. Notably, promoter activities of P*_flgBCDE_* and P*_fliC/PA1093_* were slightly but significantly lower in the PAO1 WT strain when it was cultured during Zn^2+^ stress compared to that without Zn^2+^ stress, which suggested that CzcR-based activation of both promoters failed to fully antagonize the repression caused by Zn^2+^ with the CzcR-independent pathway. This result might also account for the inhibited swimming motility as observed in the PAO1 WT strain during Zn^2+^ stress (Fig. 1E and G).

### CzcR regulated flagellar biosynthetic gene operons by directly interacting with some promoters.

Bacterial flagellar biosynthesis is controlled by numerous regulatory systems and the cyclic diguanylate (c-di-GMP) is a global bacterial second messenger that serves as a key modulator connecting flagellar motility to different regulatory pathways in P. aeruginosa and many other microbes (35–37). Since all the flagellar biosynthetic gene operons were activated by CzcR, we wondered whether flagellar gene expression and swimming motility regulated by CzcR in P. aeruginosa was mediated by the global regulatory signal c-di-GMP. To answer this, intracellular c-di-GMP contents in the PAO1 WT and Δ*czcR* strains were measured using LC-MS after they were grown in the absence or presence of Zn^2+^ stress. Unexpectedly, deletion of *czcR* did not alter the intracellular c-di-GMP content in both the presence and absence of Zn^2+^ stress (Fig. S4A). Consistently, deletion of *czcR* did not influence the expression of *cdrA,* whose expression was regarded as an indicator of c-di-GMP levels in P. aeruginosa (38, 39) (Fig. S4B). These results suggested that CzcR modulated flagellar gene expression and swimming motility of P. aeruginosa was independent of c-di-GMP.

**FIG 5:**
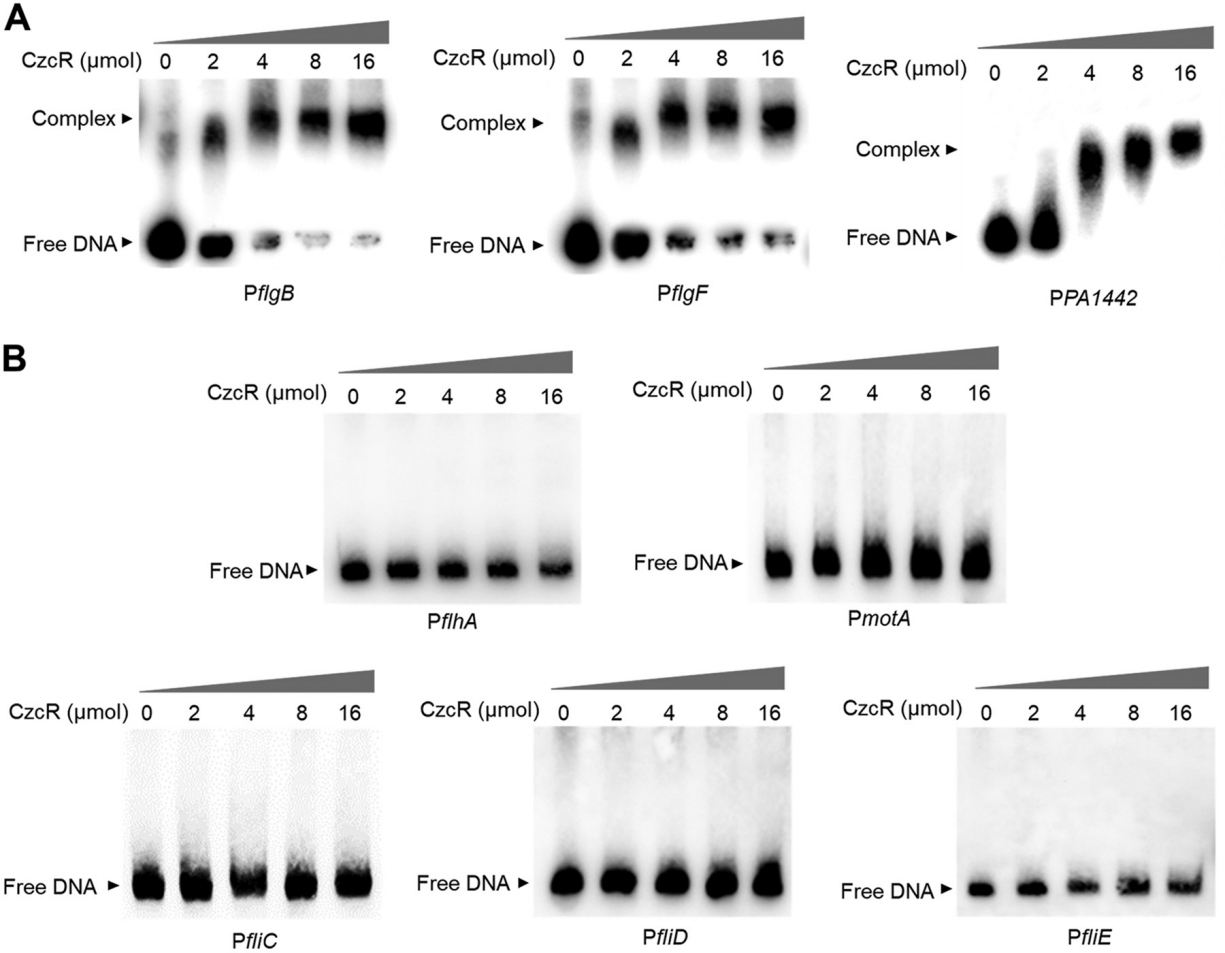
CzcR specifically binds to the promoters of operons *flgBCDE*, *flgFGHIJKL*, *PA1442/FliMNOPQR/flhB*. (A) EMSA examination showing the binding of CzcR to the promoters of *flgBCDE* (P*flgB*), *flgFGHIJKL* (P*flgF*), and *PA1442/FliMNOPQR/flhB* (P*PA1442*). (B) EMSA examination showing the incapable binding of CzcR to the promoters of *flhAF* (P*flhA*), *fliC/PA1093* (P*fliC*), *fliD* (P*fliD*), *fliEFG/PA1103/fliIJ* (P*fliE*), and *motAB* (P*motA*).

CzcR, a typical response regulator belonging to the TCS CzcS/CzcR, was predicted to contain a REC receiver domain and a linked DNA-binding Trans_reg_C domain (40). Because it was previously demonstrated to regulate PYO production by directly binding to the promoter of the *phzA1* gene (20), we then moved to explore if CzcR could recognize the flagellar biosynthetic gene promoters as well and regulate their expression directly. After the His_6_-tagged CzcR protein was purified, EMSA was performed to examine the binding potentials of CzcR with the promoters of flagellar biosynthetic genes and operons which included *flgBCDE* (P*flgB*), *flgFGHIJKL* (P*flgF*), *PA1442/FliMNOPQR/flhB* (P*PA1442*), *flhAF* (P*flhA*), *fliC/PA1093* (P*fliC*), *fliD* (P*fliD*), *fliEFG/PA1103/fliIJ* (P*fliE*), and *motAB* (P*motA*). As shown in Fig. 5A, CzcR was found to be capable of binding with the promoter sequences of *flgBCDE* (P*flgB*), *flgFGHIJKL* (P*flgF*), and *PA1442/FliMNOPQR/flhB* (P*PA1442*). As a negative control, using an irrelevant 126-bp DNA sequence from the *recA* gene did not show a CzcR-DNA complex (Fig. S5), meaning that the purified CzcR protein could specifically recognize the above promoter sequences. However, no interaction was observed between CzcR and the promoters of *flhAF* (P*flhA*), *fliC/PA1093* (P*fliC*), *fliD* (P*fliD*), *fliEFG/PA1103/fliIJ* (P*fliE*), and *motAB* (P*motA*) (Fig. 5B). These results indicated that CzcR could regulate the expression of operons *flgBCDE*, *flgFGHIJKL,* and *PA1442/FliMNOPQR/flhB* by directly interacting with their promoters but other flagellar biosynthetic genes or operons indirectly might through additional regulators.

## DISCUSSION

**FIG 6:**
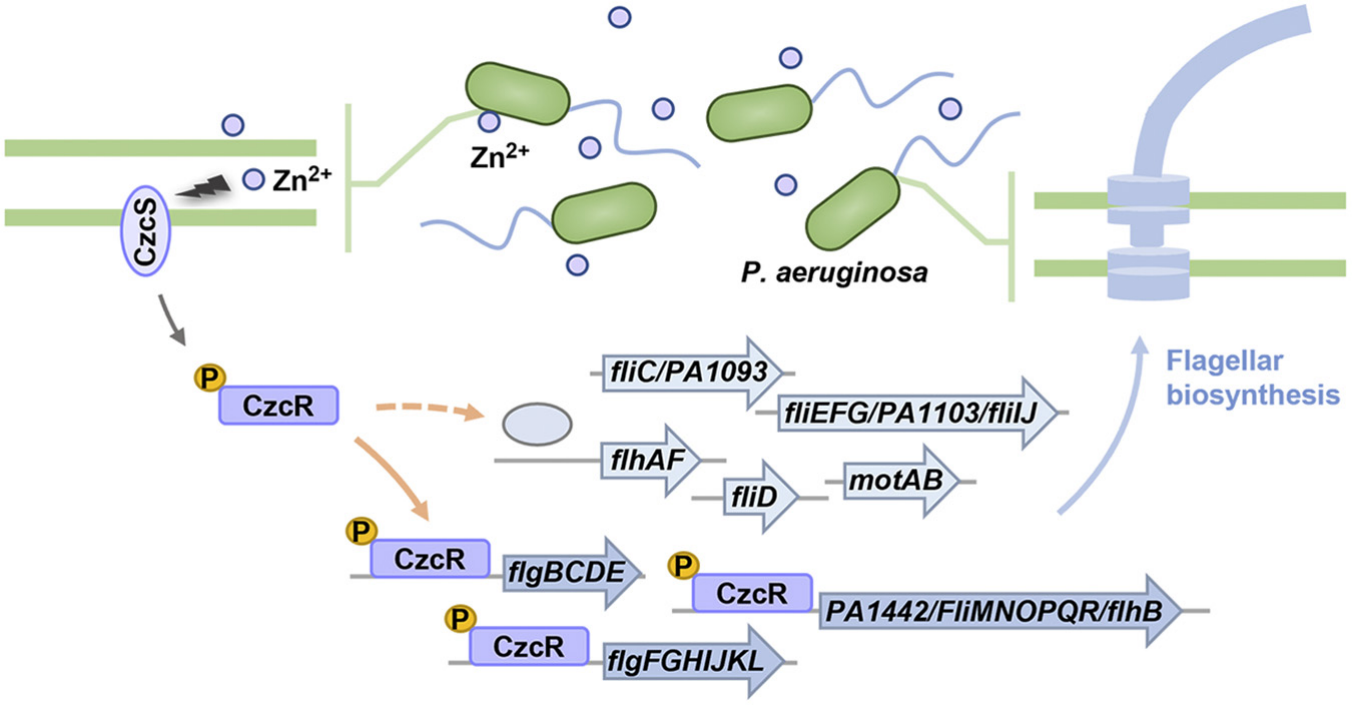
A schematic diagram illustrating the regulation of the Zn^2+^-responsive TCS CzcS/CzcR on swimming motility in P. aeruginosa. The CzcR protein is upregulated and phosphorylated to activate the expression of genes involved in flagellar biosynthesis directly (solid orange arrow) by interacting with their promoters or indirectly (dashed orange arrow) through other unknown regulators, which is essential for flagellar biosynthesis and swimming motility of P. aeruginosa during Zn^2+^ stress.

TCSs are an important group of regulatory systems in P. aeruginosa, which are employed by the pathogen to sense environmental changes and respond accordingly to maintain their ordinary growth and control production of virulence factors. However, the regulatory mechanisms and physiological roles of most TCSs are still unexplored. Characterization of these systems will facilitate the development of targeted antimicrobial strategies. CzcS/CzcR is a metal-responsive system that has been demonstrated to play important roles in modulating not only the intracellular metal homeostasis but also antibiotic resistance and quorum sensing activity (17, 20). In this study, we further reported that CzcR in this system is induced and activated during Zn^2+^ stress to upregulate expression of genes associated with flagellar biosynthesis and promote swimming motility in P. aeruginosa (Fig. 6). Indeed, it seemed that CzcR was induced and activated by Zn^2+^ to rescue the swimming motility that was abolished during Zn^2+^ stress in P. aeruginosa with unknown mechanisms.

We confirmed the role of CzcR in reducing PYO production, but loss of CzcR did not change the level of PYO production in the absence of Zn^2+^. Noticeably, unchanged PYO production between the PAO1 WT and Δ*czcR* strains was not in accordance with the previous study which reported that Δ*czcR* could produce increased PYO production even in the absence of any inducible signals, including Zn^2+^ (20). Several possibilities might result in this discrepancy. For example, this might be caused by the different growth conditions or different compositions of the culture media (41). In addition, variations in the genetic background of the PAO1 strains could be another possible factor leading to the discrepant regulatory patterns because dramatic genetic and phenotypic diversity was reported among PAO1 sublines even from the same laboratory (42). Although CzcR was activated by Zn^2+^ to repress PYO production, it was also noted that Zn^2+^ could significantly induce the PYO production without the presence of CzcR. Unfortunately, how Zn^2+^ directly or indirectly induce PYO biosynthesis remains unclear.

P. aeruginosa is a motile microorganism that is mainly driven by its polar monotrichous flagellum or type IV pili (43, 44). The swimming motility of P. aeruginosa is controlled by a number of regulatory pathways such as the signaling cascade Gac/Rsm, chemotaxis system Chp, chemosensory pathway Wsp, and the HptB pathway (45–47). Increasing studies have shown that swimming motility regulated in P. aeruginosa is broadly mediated by the global second messenger c-di-GMP (48, 49). Here, we reported that CzcR is a novel regulator which is essential for the PAO1 strain to swim during Zn^2+^ stress. It was further shown that swimming motility regulated by CzcR did not rely on the signaling molecule c-di-GMP and CzcR was able to induce the expression of genes or operons involved in flagellar biosynthesis during Zn^2+^ stress by directly interacting with some promoters or indirectly through other mediators. Indirect regulation of CzcR on these genes or operons, including *flhAF* (P*flhA*), *fliC/PA1093* (P*fliC*), *fliD* (P*fliD*), *fliEFG/PA1103/fliIJ* (P*fliE*), and *motAB* (P*motA*), might be mediated by other flagellar biosynthesis-associated regulators such as FleN and FliA owing that strong interaction of CzcR with the promoter of the operon containing *fleN* and *fliA* genes was observed (Fig. S6). Nonetheless, more detailed mechanisms await investigations.

The fluctuating concentration of transition metals is a common stress to pathogens, including P. aeruginosa, during infection (50). Metal ions such as iron, copper, and Zn^2+^ are indispensable for many biological processes in bacteria but are also detrimental to bacterial cells when in excess (51). Thus, overloading or limiting transition metals at the host-pathogen interface is a common strategy of innate immunity in human and many other mammalian hosts to combat bacterial infections (52, 53). Regarding to Zn^2+^, its accumulation within macrophages or infection sites at different tissues serves as an important line of host defense against bacterial infections (54–58). Zn^2+^ is an important transition metal ion that is associated with numerous biological processes in all kingdoms of life. Although it is known that a large percentage of proteins in bacterial cells require Zn^2+^ as their structural or catalytic factor (59), excessive Zn^2+^ exhibits toxicity by frequently competing with other metals and causing mismetallation of metalloproteins in bacteria. In the present study, we further showed that Zn^2+^ stress can inhibit expression of P. aeruginosa flagellar genes and completely abolish its swimming motility, which is known as an important virulence factor when CzcR is absent. This study indicated that the TCS CzcS/CzcR could be a promising target for precise prevention of the dissemination of P. aeruginosa using Zn^2+^-based antimicrobial agents in the future.

## MATERIALS AND METHODS

### Bacterial strains, plasmids, primers, and growth conditions.

Bacterial strains and plasmids used in this study are summarized in Table S1. Primers used in this study are summarized in Table S2. LB Broth Base and LB Agar (Invitrogen) were used to prepare medium for bacterial culture. Antibiotics were supplemented in the medium when necessary: tetracycline, 15 μg/mL; gentamicin, 20 μg/mL; kanamycin, 50 μg/mL; ampicillin, 100 μg/mL for E. coli DH5α, and tetracycline, 50 μg/mL; gentamicin, 50 μg/mL for P. aeruginosa PAO1.

### Construction of PAO1 mutants and gene complementation.

A SacB-based counterselection method was employed to knockout genes in PAO1 (60). In brief, about 600 to 1,000-bp upstream and downstream sequences flanking the target gene were amplified and cloned into the BamHI and HindIII sites of pK18mobsacB, yielding a pK18-gene plasmid for gene knockout. The pK18-gene plasmid was then delivered into P. aeruginosa PAO1 via triparental mating with the help of the E. coli pRK2013 strain. Recovered colonies were selected on LB agar plates containing 10% sucrose. Mutants were confirmed by colony PCR and sanger sequencing. Complementation assay for *czcR* was achieved by the mini-Tn7 system (61). Briefly, sequence of the *czcR* coding region with a His tag and its native promoter was amplified from the genomic DNA of PAO1 and cloned into the BamHI and HindIII sites located on the pUC18T-mini-Tn7T-Gm plasmid, generating the pTn7-*czcR* plasmid. pTn7-*czcR* was then integrated into PAO1 genome via coelectroporation with the helper plasmid pTNS2.

### Construction of promoter-*lux* reporter strains and measurement of promoter activity.

Mini-CTX-*lux* system was used to measure promoter activities (62). Promoter regions of target genes were amplified from PAO1 genome and cloned into the KpnI and BamHI sites on the mini-CTX-*lux* plasmid. The resultant plasmids were integrated into PAO1 genome by biparental mating. Luminescence was measured to indicate the activity of corresponding promoters. Briefly, overnight culture of each strain carrying the promoter-*lux* fusions was adjusted to OD_600_ of 1.0 and then 1:100 diluted into 2 mL fresh LB medium with or without the supplementation of 0.5 mM ZnSO_4_. Luminescence was monitored after 8 h growth at 37°C in a microplate reader (BioTek).

### Pyocyanin production assays.

Pyocyanin production measurement was performed as previously described (63). The overnight culture was adjusted to the OD_600_ of 1.0 and then 1:100 diluted into 2 mL fresh LB medium with or without the supplementation of 0.5 mM ZnSO_4_. A 1.5 mL culture was collected every 2 h during growth and centrifuged at 13,000 rpm for 2 min. A 1 mL supernatant was transferred to a 24-well plate and the absorbance at A_695_ was measured in a microplate reader (BioTek).

### Swimming motility assay.

The plates used for swimming assay was prepared as follows: 10 g/L bacto-peptone, 5 g/L NaCl with 0.2% (wt/vol) bacto-agar. The overnight culture was adjusted to the OD_600_ of 1.0 and 2 μL cell culture was inoculated on the center of the swimming plates with or without the supplementation of 0.5 mM ZnSO_4_. The plates were incubated at 37°C for 14 h and diameters of the swimming zones were measured.

### RNA extraction and quantitative real-time PCR.

Overnight culture of PAO1 strains was 1:100 diluted into 2-mL fresh LB medium with or without the supplementation of 0.5 mM ZnSO_4_ and grown for 8 h. One mL bacterial culture was harvested, and total RNA were extracted using the RNA extraction kit (Omega) following the manufacturer’s instructions. cDNA was reverse transcribed using the TranScript one-step gDNA removal and cDNA Synthesis kit (TransGen Biotech). qPCR was performed using the 2 × SYBR green Master Mix Reagent (Yeasen) in an ABI QuantStudioTM6 Flex system (Applied Biosystems). The *recA* gene was selected as the internal reference control and the 2^-ΔΔCt^ method was used to calculate the relative expression of the target genes (64). The result was displayed as the mean of biological triplicates.

### Western blotting.

Overnight culture of the Δ*czcR* mutants with complementation of His_6_-tagged *czcR* or *czcR* variants (D8A and D51A) was 1:100 diluted into 5-mL fresh LB medium and subcultured with or without the supplementation of 0.5 mM ZnSO_4_ until OD_600_ was 1.0. Approximately 2.0 × 10^9^ cells were harvested and lysed by 40 μL xTractor Buffer (TaKaRa) supplemented with protease inhibitor. After centrifugation, supernatant was mixed with SDS-loading buffer. Proteins were separated by SDS-PAGE and transferred to polyvinylidene difluoride (PVDF) membranes by electroblotting. Membranes were blocked with Blocking buffer (5% nonfat milk PBST) followed by immunoblotting using anti-His_6_ antibody (Abcam) and horseradish peroxidase-conjugated goat anti-rabbit antibody (TransGen Biotech). Proteins were detected using the ECL kit (Bio-Rad) according to the manufacturer’s protocol.

### Transmission electron microscopy.

P. aeruginosa PAO1 WT and Δ*czcR* strains were harvested after they were cultured on the center of agar plates (10 g/L bacto-peptone, 5 g/L NaCl, 0.5% [wt/vol] bacto-agar) with or without the supplementation of 0.5 mM ZnSO_4_. Cells were spread in a drop of MilliQ H_2_O and covered with Parlodion (Mallinckrodt) carbon-coated grid (300 mesh). Then, the cells were fixed with 0.5% uranyl acetate. Samples were examined by the Field Emission Transmission Electron Microscopy (Talos F200S) and flagellar length was measured by Image J (version: 1.52v).

### Protein purification and electrophoretic gel mobility shift assay (EMSA).

Protein purification was performed as previously described with slight modifications (65). The *czcR* coding region was amplified from the PAO1 genome and cloned into the BamHI and XhoI sites on the pET28a vector, generating pET28a-*czcR*. The plasmid was transformed into E. coli BL21(DE3) and the strain containing pET28a-*czcR* was incubated until OD_600_ of 0.6~0.8. Expression of the His_6_-tagged CzcR was induced with 0.5 mM isopropyl-β-d-thiogalactoside (IPTG) at 18°C for 16 h. Purification of the His_6_-tagged CzcR protein was conducted using a Ni^2+^-affinity column. For EMSA, promoter sequences were amplified by PCR using the primers in Table S2 from the PAO1 genome and tagged with biotin using a DNA labeling kit (Thermo Scientific). The EMSA kit (Thermo Scientific) was used to test the interaction of CzcR with promoter sequences according to the manufacturer's instructions.

### c-di-GMP measurement.

Quantifications of c-di-GMP in P. aeruginosa strains was performed as described previously with modifications (37). In brief, P. aeruginosa overnight culture was 1:100 diluted and incubated in LB medium containing 0.5 mM ZnSO_4_ or not for 8 h. One mL cell culture was harvested, and cells were lysed with perchloric acid (70% vol/vol). After neutralization with 2.5 M KHCO_3_, supernatants were collected for c-di-GMP measurement by liquid chromatography-mass spectrometry (LC-MS). LC-MS was performed using a Q Exactive Focus Hybrid Quadrupole-Orbitrap mass spectrometer (Thermo Fisher Scientific) with a 1.8 μm, 100 × 2.1 mm high-strength silica (HSS) T3 column (Waters). The *m/z* 691 > 248 transition was used for quantification.

### Statistical analysis.

Experimental data were analyzed by either Student's *t* test or one-way analysis of variance (ANOVA) as indicated in the figure legends using GraphPad Prism software (version 8). Differences with *P < *0.05 were considered statistically significant. *, **, and *** indicated *P < *0.05, *P < *0.01, and *P < *0.001, respectively.
