# Supplementary material for: CzcR Is Essential for Swimming Motility in Pseudomonas aeruginosa during Zinc Stress
Source: Microbiol Spectr. 2022 Nov 23;10(6):e02846-22. doi: 10.1128/spectrum.02846-22 (PMC9769499; doi:10.1128/spectrum.02846-22)
Supplement: Supplemental file 1 — Supplemental material. Download spectrum.02846-22-s0001.pdf, PDF file, 0.4 MB [file spectrum.02846-22-s0001.pdf]

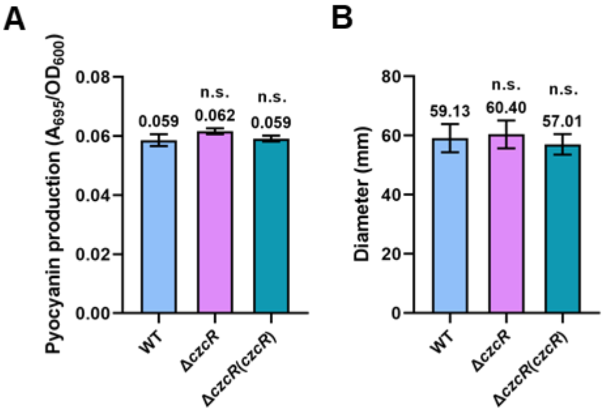

Figure S1. Quantification of PYO production (A) and swimming motility (B) with the supplementation of Na_2SO_4 in PAO1 WT, Δ*czcR* and Δ*czcR*(*czcR*) strains. n.s.: not significant *versus* WT based on one-way ANOVA.

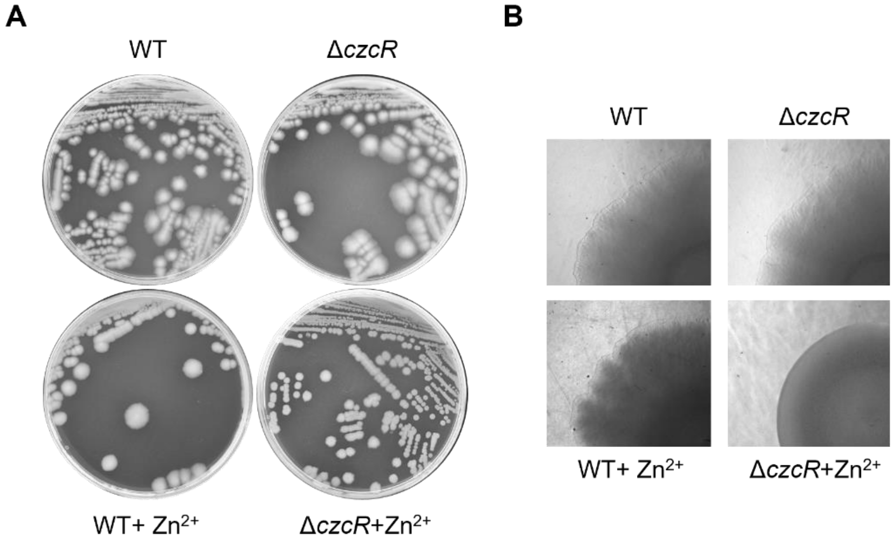

Figure S2. Colony phenotypes of PAO1 WT and Δ*czcR* strains grown on the plates with the supplementation of Zn^{2+} or not. (**A**) PAO1 WT and Δ*czcR* strains were streaked on the LB agar plates with the supplementation of Zn^{2+} or not. (**B**) Colonies of PAO1 WT and Δ*czcR* strains were observed under a stereo microscope (Laite LSF763). The Δ*czcR* strain grown in the presence of Zn^{2+} stress showed a smooth colony phenotype compared to others.

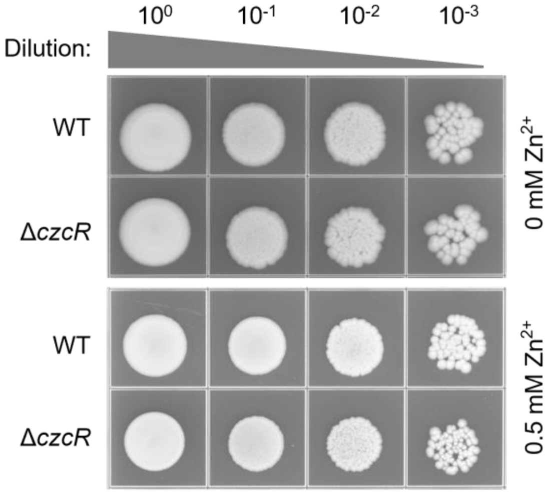

Figure S3. Growth of the PAO1 WT and Δ*czcR* strains on the plates with or without Zn^{2+} supplementation. Overnight culture of PAO1 WT and Δ*czcR* strains were serial diluted and spotted on the LB plates containing Zn^{2+} or not, which showed comparable growth between WT and Δ*czcR* strains in the absence or presence of Zn^{2+} stress.

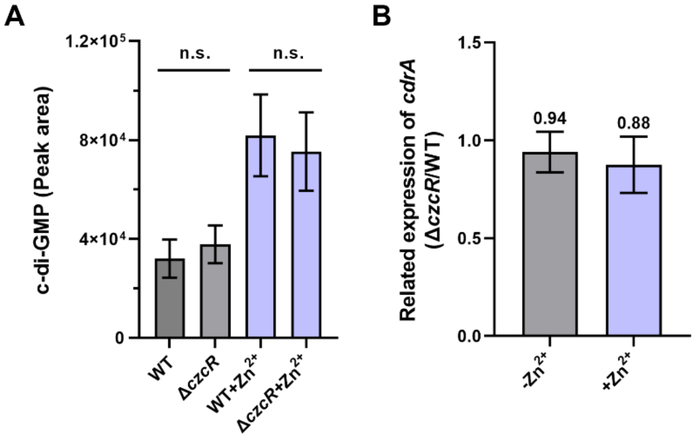

Figure S4. CzcR does not affect the c-di-GMP signaling pathway. (**A**) Measurement of intracellular c-di-GMP by LC-MS in PAO1 WT and Δ*czcR* strains grown in the absence or presence of Zn^{2+} stress. (**B**) Measurement of the *cdrA* expression by RT-qPCR in PAO1 WT and Δ*czcR* strains grown in the absence or presence of Zn^{2+} stress. n.s. not significant, based on Student's *t* test.

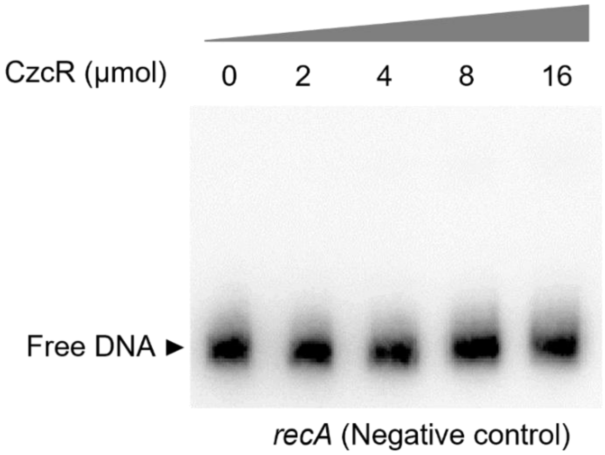

Figure S5. CzcR does not bind to the DNA fragment amplified from the *recA* gene. EMSA examination showing the incapable binding of CzcR to a 126-bp DNA fragment which served as a negative control.

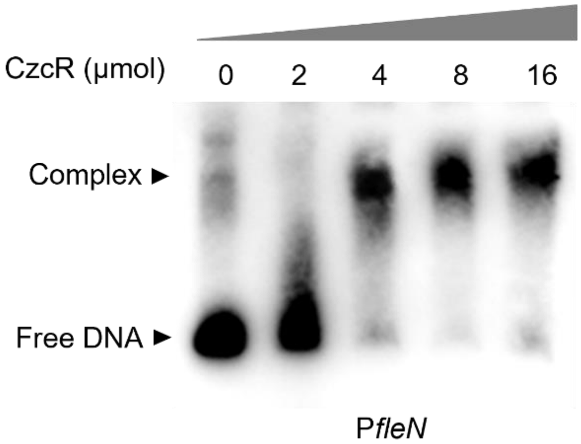

Figure S6. CzcR binds to the promoter of the operon containing the *fleN* and *fliA* genes. EMSA examination showing the binding of CzcR to the promoter of *fleN/fliA* (P*fleN*).

Table S1. Bacterial strains and plasmids used in this study.

**Strains and plasmids**	**Description**	**Reference or source**
**Strains**		
P. aeruginosa PAO1	Wild-type PAO1	Lab collection
Δ*czcR*	PAO1 with the deletion of *czcR*	This study
Δ*czcR*(*czcR*)	Δ*czcR* with the complemented *czcR*	This study
Δ*czcR*(*czcR*^{D8A})	Δ*czcR* with the complemented *czcR*^{D8A}	This study
Δ*czcR*(*czcR*^{D51A})	Δ*czcR* with the complemented *czcR*^{D51A}	This study
E. coli DH5α	F-φ80d lacZΔM15 Δ(lacZYA-argF) U169 end A1 recA1 hsdR17 (rk$^-$, mk$^+$) supE44λ- thi-1 gyrA96relA1 phoA	TransGen Biotech
E. coli BL21 (DE3)	F$^-$ *ompT* *hsd*S($r_B^- m_B^-$) *gal dcm*(DE3)	TransGen Biotech
**Plasmids**		
p18Gm-Δ*czcR*	For *czcR* deletion	This study
pRK2013	Helper plasmid for the tri-parental matting, Kan^r	Lab collection
CTX-P$_{flgB}$	mini-CTX-lux containing the promoter of *flgB*	This study
CTX-P$_{flgF}$	mini-CTX-lux containing the promoter of *flgF*	This study
CTX-P$_{fliC}$	mini-CTX-lux containing the promoter of *fliC*	This study
CTX-P$_{fliD}$	mini-CTX-lux containing the promoter of *fliD*	This study
CTX-P$_{fliE}$	mini-CTX-lux containing the promoter of *fliE*	This study
CTX-P$_{PA1442}$	mini-CTX-lux containing the promoter of *PA1442*	This study
CTX-P$_{flhA}$	mini-CTX-lux containing the promoter of *flhA*	This study
CTX-P$_{motA}$	mini-CTX-lux containing the promoter of *motA*	This study
pTNS2	Helper plasmid for the Tn7 transposition, Amp^r	Lab collection
pTn7-*czcR*	pUC18T-mini-Tn7T-Gm containing the promoter region and ORF of *czcR*	This study
pTn7-*czcR*^{D8A}	Point mutated of D8A in pTn7-czcR vector	This study
pTn7-*czcR*^{D51A}	Point mutated of D51A in pTn7-czcR vector	This study
pET28a-*czcR*	pET-28a (+) containing the ORF of *czcR*	This study

Table S2. Primers used in this study.

**Name**	**Sequence (5'-3')**	**Description**
pK-*czcR*-uF	GAGCTCGGTACCCGGGGATCCTGTCCTCGACGCTCCAGGA	Amplify the homologous sequences for *czcR* deletion
pK-*czcR*-uR	CGTCATGTTCGCCCCTATATAAAGTATGGATT	
pK-*czcR*-dF	ATATAGGGGCGAACATGACGCCACCAGGCCGA	
pK-*czcR*-dR	ACGACGGCCAGTGCCAAGCTTGGAGAACTGCGAGAGCTGGC	
Veri-*czcR*-F	CCAGTTGGGTCCAGGCGATCTC	Verify the deletion of *czcR* in PAO1
Veri-*czcR*-R	CAGTTCCTCGGCATTGGACT	
CTX-P*flgB*-F	CTATAGGGCGAATTGGGTACCGAATACGATGCGATGTTCGAGG	Amplify the promoter of P*flgB*
CTX-P*flgB*-R	CTAAAGAAGAATTGGGGATCCGTTGTTGGCCAGCACTTCGG	
CTX-P*flgF*-F	CTATAGGGCGAATTGGGTACCCCAATGCCAAGACCATCCAG	Amplify the promoter of P*flgF*
CTX-P*flgF*-R	CTAAAGAAGAATTGGGGATCCGTTGTTGGCATGAGCACGC	
CTX-P*fliC*-F	CTATAGGGCGAATTGGGTACCGAATCCGGGTTTTTCTCGAAC	Amplify the promoter of P*fliC*
CTX-P*fliC*-R	CTAAAGAAGAATTGGGGATCCTGGTCAGACGCTGCAACGA	
CTX-P*fliD*-F	CTATAGGGCGAATTGGGTACCGCAACTTGAACTTCAGCATCGA	Amplify the promoter of P*fliD*
CTX-P*fliD*-R	CTAAAGAAGAATTGGGGATCCCATCTTGTTCACCAGATCGGTG	
CTX-P*fliE*-F	CTATAGGGCGAATTGGGTACCCTGCGCTACAAGCTCGCC	Amplify the promoter of P*fliE*
CTX-P*fliE*-R	CTAAAGAAGAATTGGGGATCCGGCTTCCATTTGCATGGAAC	
CTX-P*flhA*-F	CTATAGGGCGAATTGGGTACCGATTACGCTAGCTCATCGGGTT	Amplify the promoter of P*flhA*
CTX-P*flhA*-R	CTAAAGAAGAATTGGGGATCCCAGGTTGCTGCGCACATTG	
CTX-P*motA*-F	CTATAGGGCGAATTGGGTACCGGATGGACAACGCCAGCG	Amplify the promoter of P*motA*
CTX-P*motA*-R	CTAAAGAAGAATTGGGGATCCGGAGAGCAGGAAGCCCCC	
CTX-P*PA1442*-F	CTATAGGGCGAATTGGGTACCGATGAGGAAACCCTCGCCG	Amplify the promoter of P*PA1442*
CTX-P*PA1442*-R	CTAAAGAAGAATTGGGGATCCCAGCTTCAGCTTGCTCTTGCC	
Tn7-*czcR*-F	CATGAGCTCACTAGTGGATCCCGGCATGTTCCGCTCCTCGTC	Amplify *czcR* to construct pTn7-*czcR*
Tn7-*czcR*-R	CGAGGTACCGGGCCCAAGCTTTCACGGCGCGCTTCCAGGACG	
Tn7-D8A-F	TATCGAAGCGGAAGTCAAGACTGCCGACTACCTG	For the D8A mutation of *czcR*
Tn7-D8A-R	TGACTTCCGCTTCGATAATAAGGATGCGCATG	
Tn7-D51A-F	TGATCCTCGCGGTCAACCTGCCCGGCATCGAC	For the D51A mutation of *czcR*
Tn7-D51A-R	GTTGACCGCGAGGATCACCAGTTCGTAAGGGT	
PTn7R	CACAGCATAACTGGACTGATTTC	Verify the Tn7-based chromosomal integration
PglmS-down	GCACATCGGCGACGTGCTCTC	
28a-*czcR*-F	CAGCAAATGGGTCGCGGATCCATGCGCATCCTTATTATCGAAG	Amplify *czcR* to construct pET28a-*czcR*
28a-*czcR*-R	TGGTGGTGCTCGAGTGCGGCCGCTCATCGGCGCGCTTCCAGG	
Veri-28a-F	GAATTGTGAGCGGATAACAATTCC	Verify the construction of pET28a-*czcR*
Veri-28a-R	CAAGACCCGTTTAGAGGCCC	
P_{recA}-F	TCCATTGAAGTCCTCGCGAAGT	Amplification of DNA sequences for EMSA
P_{recA}-R	GCAACCGTTCGGAACATTCTTCC	
P_{flgB}-F	GTTCCAGGGGCTTGCCAC	
P_{flgB}-R	TCGAAACTGATGCTCATGGCTG	

P*flgF*-F	TTGCCCCCGAAGCCTCCGAAAG
P*flgF*-R	GACGTACAGCATCTTGTCCAT
P*fliC*-F	TTCAGGACCGATATTGGCGAGT
P*fliC*-R	GGTGATTTCCTCCAAAGGACCTAT
P*fliD*-F	TACACAGTGTCCTGTTCGAAACCCG
P*fliD*-R	TACTCCTATCGAGATACCGGCCA
P*fliE*-F	CTGTTCGAGCGTGCGC
P*fliE*-R	CTGACTCATGACTCTTCCTCCAACAG
P*PA1442*-F	TTTCTTAGCCATGCCAAAAATCCGT
P*PA1442*-R	GGCAATTTCGCAAAAGACGGG
P*fleN*-F	CATCTGCTTCATACCTTGTGTTGTCG
P*fleN*-R	TGAGGACGTGGGAAGAACCG
P*flhA*-F	TTGGCGAAAACCCTTTTCAATCAATGAATT
P*flhA*-R	GCGCACATTGCTGATCAGTTG
P*motA*-F	GCCGATGATTTTTGACATGAGGACC
P*motA*-R	TCATGGGTGTCGGGCTCG
